# Determining the number of stimuli required to reliably assess corticomotor excitability and primary motor cortical representations using transcranial magnetic stimulation (TMS): a protocol for a systematic review and meta-analysis

**DOI:** 10.1186/s13643-015-0095-2

**Published:** 2015-08-11

**Authors:** Rocco Cavaleri, Siobhan M. Schabrun, Lucy S. Chipchase

**Affiliations:** Brain Rehabilitation and Neuroplasticity Unit, School of Science and Health, The University of Western Sydney, Sydney, New South Wales 2560 Australia

**Keywords:** Transcranial magnetic stimulation, Reliability, Motor cortex, Cortical reorganisation, Systematic review

## Abstract

**Background:**

Transcranial magnetic stimulation (TMS) is a technique that can be used to assess corticospinal plasticity. Current TMS practices involve the administration of multiple stimuli over target areas of the participant’s scalp. However, these procedures require 1 to 2 h per assessment. Decreasing the number of stimuli delivered during TMS assessments would improve time efficiency and decrease participant demand. Thus, the aim of this review is to determine the number of TMS stimuli required to reliably measure (1) corticomotor excitability to a target muscle at a single cranial site and (2) the topography of the primary motor cortical representation for a target muscle across multiple cranial sites (termed ‘mapping’).

**Methods/design:**

A systematic review and meta-analysis will be conducted. Electronic databases will be searched using pre-determined search terms to identify relevant studies and evaluate the studies for inclusion and risks of bias. Two independent reviewers will extract the data. Any disagreements will be resolved by a third reviewer. Studies employing single-pulse TMS to measure (1) corticomotor excitability at a single cranial site or (2) the topographic cortical organisation of a target muscle across a number of cranial sites, published before May 2015, will be included if they meet the eligibility criteria. Outcomes will include motor-evoked potential amplitude, map volume, number of active map sites, location of the map centre of gravity, and distance between the centres of gravity of the target muscle and one or more neighbouring muscles.

**Discussion:**

To our knowledge, this review will be the first to systematically explore the number of TMS stimuli required to reliably measure both corticomotor excitability and the topography of primary motor cortical representations. This research has the capacity to improve the efficiency of TMS, decrease participant demand, and facilitate the use of TMS as an outcome measurement tool in clinical populations.

**Systematic review registration:**

PROSPERO CRD42015024579

**Electronic supplementary material:**

The online version of this article (doi:10.1186/s13643-015-0095-2) contains supplementary material, which is available to authorized users.

## Background

First described by Barker, Jalinous, and Freeston in 1985, transcranial magnetic stimulation (TMS) is a non-invasive, safe, and painless technique used to stimulate the human brain [1]. Initially, TMS was used to investigate central nervous system integrity among neurological populations [1]. However, over the last 15 years, TMS has evolved to include a variety of broader applications. Such applications range from pre-surgical tumour mapping to exploration of cortical reorganisation in pathological populations [2, 3]. Transcranial magnetic stimulation has also been shown to be a valuable tool for predicting functional recovery and investigating the neurophysiological effects of various rehabilitation approaches [4, 5]. However, the time-consuming nature of TMS limits its applicability in clinical practice [3].

During TMS, a stimulation coil introduces a magnetic field over the target area of the participant’s scalp, inducing a secondary electrical current within cortical tissue [6]. In the case of the motor cortex, the electrical current triggers action potential propagation down the corticospinal tract to contralateral peripheral motor neurons, eliciting a motor response [7]. Electrical potentials generated by muscle cells following motor cortex stimulation are measured as motor-evoked potentials (MEPs) using electromyography. Changes in MEPs over time suggest alterations to the structure and function of a participant’s neuronal network [6, 7].

Current TMS practices involve the administration of multiple stimuli over target areas of the participant’s scalp [3, 8]. Depending on the desired neurophysiological index, stimuli are either applied at a single cranial site, or systematically over a predefined grid, in a process known as mapping. Stimuli are delivered as single pulses, typically with 3 to 5 s between each stimulus [3]. During mapping, four to five stimuli are commonly administered to each cranial site [3, 8]. Single-site analyses at the optimal site, known as the ‘hotspot’, typically utilise ten stimuli [9, 10]. Recording the mean outcome of repeated trials is thought to enhance reliability and enable identification of potential outliers [11]. Although these TMS procedures have been validated using magnetic resonance imaging (MRI), the International Federation of Clinical Neurophysiology highlights that current practices are inefficient and that recommendations were determined arbitrarily [6].

Administering five stimuli per cranial site to map the cortical representation of a particular muscle requires 1 to 2 h per assessment, restricting its use beyond the research environment [12]. Further, prolonged TMS assessments have been associated with significant increases in participant fatigue and discomfort [6]. This becomes particularly important when investigating neurological populations, where increased metabolic demands and poor baseline levels of endurance may limit adherence [13]. Similarly, prolonged assessments are impractical when exploring conditions involving chronic pain [12]. The importance of reducing data collection time is also evident from the observation that corticospinal excitability fluctuates with participant arousal and concentration, which may vacillate throughout prolonged investigations [14].

Reducing the number of stimuli delivered to each cranial site during TMS has the potential to improve the efficiency of the procedure while decreasing participant demand [3]. However, lowering the number of stimuli per site may also compromise the procedure’s reliability [6]. No reviews have systematically examined the reliability of outcomes obtained using varying numbers of stimuli per cranial site during TMS [6]. Here, we present the protocol for a review that aims to determine the number of TMS stimuli required to reliably measure (1) corticomotor excitability to a target muscle at a single cranial site and (2) the topography of the primary motor cortical representation for a target muscle across multiple cranial sites (termed ‘mapping’).

## Methods/design

This protocol was prepared in accordance with the Preferred Reporting Items for Systematic Review and Meta-Analysis Protocols (PRISMA-P) [15] [see Additional file 1] and a measurement tool to assess systematic reviews (AMSTAR) [16]. The protocol has been registered with the International Prospective Register of Systematic Reviews (PROSPERO; registration number CRD42015024579).

### Review question

How many TMS stimuli are required to reliably measure (1) corticomotor excitability to a target muscle at a single cranial site and (2) the topography of the primary motor cortical representation for a target muscle across multiple cranial sites?

### Search strategy

The methods for this systematic review were developed using items from Chapter 13 of the Cochrane Handbook for Systematic Reviews relevant to the reporting of systematic reviews of diagnostic reliability studies [17]. The search strategy was developed in consultation with a librarian with systematic review expertise.

Searches will be conducted in CINAHL, CENTRAL, EMBASE, MEDLINE, Neuroscience Information Framework (NIF), PEDro, PsycINFO, PubMed, Scopus, and Web of Science databases. No limits will be placed upon language or location of publication. Where necessary, articles will be translated to English by an independent interpreter for analysis. Keywords and medical subject headings (MeSH) related to transcranial magnetic stimulation, cortical reorganisation, and reliability will be used. The terms ‘transcranial magnetic stimulation’, ‘cortical reorganisation’, and ‘reliability’ will be used in varying combinations to identify relevant literature. Search strategies will be customised to suit each database [see Additional file 2].

#### Other resources

Authors will contact experts in the field of TMS and search Google Scholar for additional studies. Searches of the International Clinical Trials Registry Platform (ICTRP) will be conducted to identify recently completed studies. The reference lists of all relevant articles will be analysed to identify additional trials. Studies from these sources satisfying the eligibility criteria will be included in the systematic review.

### Types of participants

To reflect the widespread applicability of TMS, no restrictions will be placed upon participant age or gender. Both healthy and clinical populations will be eligible for inclusion.

### Inclusion criteria

1. Full-text articles published prior to May 2015.
2. Diagnostic reliability studies comparing the effect of the number of TMS stimuli on the within- or between-session reliability of either (a) corticomotor excitability at a single cranial site or (b) the topographic cortical organisation of a target muscle across multiple cranial sites. Only studies employing single-pulse TMS will be included. The number and position of targeted cranial coordinates are required to be consistent within and between sessions.

### Exclusion criteria

1. Journal or conference abstracts with no associated full-text article
2. Studies not exploring within- or between-session reliability
3. Studies investigating within- or between-session reliability of corticomotor excitability or topographic cortical representation where the number of TMS stimuli is not varied
4. Studies comparing the reliability of TMS outcomes obtained between cortical hemispheres, rather than within or between sessions using the same hemisphere
5. Studies reporting only the ‘ideal’ number of stimuli at a particular site
6. Studies presenting reliability predictions based upon mathematical models, rather than experimental testing, as mathematical models are unable to take into account inter-individual fluctuations in corticospinal activity [18]
7. Studies investigating the effects of interventions.
8. Studies employing repetitive TMS to induce, rather than measure, neuroplastic changes.

### Outcomes

For investigations of corticomotor excitability at a single cranial site, eligible studies must report motor-evoked potential amplitude. For investigation of the topographic representation (map) of a target muscle across multiple cranial sites, eligible studies must report at least one of the following: motor-evoked potential amplitude, map volume, number of active map sites, location of the map centre of gravity (CoG), or distance between the centres of gravity of the target muscle and one or more neighbouring muscles. These indices represent those most commonly assessed during TMS studies [6, 19].

### Data management

Search results will be exported to EndNote citation software for automatic duplicate removal. Any duplicates overlooked by the programme will be manually removed. Two independent reviewers will then screen the exported articles for relevance by title and abstract. Potentially relevant papers will be retrieved as full-text articles and assessed according to the eligibility criteria by these two reviewers. An additional reviewer will be consulted should there be any uncertainty or disagreement regarding the eligibility of studies. Excluded studies and reasons for exclusion will be recorded.

### Data extraction

Participant characteristics, sample sizes, TMS protocols, neuroplastic indices, and reliability measurements will be extracted by two independent reviewers. Any disagreements will be resolved by a third reviewer. If data are missing, authors will be contacted a maximum of three times, after which the data will be considered irretrievable.

Where studies obtain outcomes in response to multiple TMS stimulation intensities, the intensity closest to 120 % of resting motor threshold (rMT) will be analysed as this value has been shown to be valid and reliable over time [20]. Additionally, 120 % of rMT is the most commonly employed stimulation intensity during TMS investigations [21]. Similarly, in studies using active motor thresholds (aMTs), the analysis closest to 10 % of maximal voluntary contraction will be included [20, 21].

### Assessment of methodological quality

Two tiers of methodological quality assessment will be performed. First, the general experimental design of each study will be appraised using a custom appraisal tool. Second, the included studies will be assessed according to the TMS-specific checklist developed by Chipchase et al. [22].

#### General experimental design

A custom quality appraisal tool will be utilised to assess general methodological quality [see Additional file 3]. The tool will consist of relevant items from both the Quality Assessment of Reliability Studies (QAREL) [23] checklist and the guidelines for assessing reliability studies developed by Bialocerkowski et al. [24]. These appraisal tools have been shown to possess good inter-rater reliability [25]. Additionally, they provide insight into both internal and external validity [24, 25].

Two reviewers will independently assess studies that satisfy the eligibility criteria. Items will be scored as either present (1) or absent (0), and a score out of 11 will be obtained via summation. Studies will not be excluded based upon quality. In accordance with QAREL-based recommendations provided by Triano et al. [26], studies scoring four or less will be deemed to be of low quality, scores between five and seven will be classified as moderate quality, and studies scoring eight or above will be classified as high quality. Any disagreement will be resolved by a third reviewer.

#### TMS-specific protocol and reporting

The TMS checklist designed by Chipchase et al. [22] will be used to assess TMS-specific elements of methodological and reporting quality [see Additional file 4]. This checklist was produced via a two-round international Web-based Delphi consensus study. Appraising the TMS protocol itself will enable identification of methodological shortcomings that would not be revealed during assessments of general experimental design. Items will be marked ‘Yes’ or ‘No’ by two independent reviewers based upon whether or not requirements for the corresponding item have been satisfied. Disagreements will be resolved by a third reviewer.

### Strategy for data synthesis

Reliability estimates in the form of intraclass correlation coefficients (ICCs) will be pooled using StatsDirect 3 Software. The intraclass correlation coefficient is the most accurate and commonly used indication of the size and direction of association between two variables [27]. Intraclass correlation coefficients for within- and between-session reliability will be interpreted using the following values: less than 0.50 = poor, 0.50 to 0.65 = moderate, 0.65 to 0.80 = good, and greater than 0.80 = excellent [27]. Confidence intervals surrounding ICCs will be calculated using StatsDirect software, and significance will be set at *p* < 0.05.

A random-effects model will be used during analyses, as methodological heterogeneity is inevitable during manual TMS assessments [17]. The impact of heterogeneity will be calculated using the *I*^2^ statistic and interpreted as follows: 0 to 40 % may be unimportant, 30 to 60 % may represent moderate heterogeneity, 50 to 90 % may represent substantial heterogeneity, and 75 to 100 % represents considerable heterogeneity [17]. In cases of substantial methodological or statistical heterogeneity, subgroup analyses will be performed to highlight potential reasons for this heterogeneity. If, following subgroup analyses, substantial statistical heterogeneity remains, results will be synthesised descriptively, rather than statistically.

### Analysis of subgroups or subsets

Following initial meta-analyses, comprehensive subgroup analyses will be performed to investigate the influence of methodological inconsistencies and participant variability. Subgroup analyses will be performed according to participant age (greater than or less than 65 years), target region (facial, upper limb, or lower limb musculature), test conditions (active or resting target musculature), population (clinical or healthy), and intersession interval (greater than 72 h or less than 72 h). Additional analyses will also be performed with low-quality studies excluded to determine if general experimental design quality significantly influences the results.

## Discussion

To our knowledge, this review will be the first to systematically explore the number of TMS stimuli required to reliably measure (1) corticomotor excitability to a target muscle at a single cranial site and (2) the topography of the primary motor cortical representation for a target muscle across multiple cranial sites. This research has the capacity to improve the efficiency of TMS, decrease participant demand, and facilitate the use of TMS as an outcome measurement tool in clinical populations. The review will also facilitate further research into this area, identifying potential areas for development.

### Limitations

As database searches will be limited to full-text articles, bias may be introduced through the exclusion of data in grey literature. This ‘publication bias’ may inflate reliability estimates, as studies with desirable or significant results are more likely to be granted publication [17]. Additionally, including only one stimulation intensity per study may reduce the external validity of this review.

### Ethics and dissemination

This review does not require ethical approval. Results will be presented at scientific meetings and published in peer-reviewed journals. All publications and presentations related to the study will be authorised and reviewed by the study investigators.

### Review status

The reviewers have commenced searching relevant studies on the electronic databases. This review is expected to be complete by July 2015.

## Additional files

Additional file 1:
**PRISMA-P (Preferred Reporting Items for Systematic review and Meta-Analysis Protocols) 2015 checklist.** Recommended items to address in a systematic review protocol. (DOC 81 kb) Additional file 2:
**Search strategies.** Detailed search strategies that will be utilised to conduct the proposed systematic review. (DOCX 16 kb) Additional file 3:
**Custom methodological quality checklist.** Checklist that will be utilised to assess the general experimental design of included studies. (DOCX 15 kb) Additional file 4:
**TMS-specific methodology checklist.** Checklist that will be utilised to assess the TMS-specific methodological and reporting quality of included studies. (DOCX 15 kb)

## Abbreviations

AMSTAR: a measurement tool to assess systematic reviews
CoG: centre of gravity
ICC: intraclass correlation coefficient
MEP: motor-evoked potential
MOOSE: Meta-analysis of Observational Studies in Epidemiology
PRISMA-P: Preferred Reporting Items for Systematic Reviews and Meta-analyses
QAREL: Quality Assessment of Reliability Studies
QUADAS-2: Quality Assessment of Diagnostic Accuracy Studies
TMS: transcranial magnetic stimulation
