# Supplementary material for: Determining the number of stimuli required to reliably assess corticomotor excitability and primary motor cortical representations using transcranial magnetic stimulation (TMS): a protocol for a systematic review and meta-analysis
Source: Syst Rev. 2015 Aug 11;4:107. doi: 10.1186/s13643-015-0095-2 (PMC4531429; doi:10.1186/s13643-015-0095-2)
Supplement: Additional file 3: — Custom methodological quality checklist. Checklist that will be utilised to assess the general experimental design of included studies. (DOCX 15 kb) [file 13643_2015_95_MOESM3_ESM.docx]

| Study | Item 1 | Item 2 | Item 3 | Item 4 | Item 5 | Item 6 | Item 7 | Item 8 | Item 9 | Item 10 | Item 11 | Total /11 |
| --- | --- | --- | --- | --- | --- | --- | --- | --- | --- | --- | --- | --- |
|  |  |  |  |  |  |  |  |  |  |  |  |  |
|  |  |  |  |  |  |  |  |  |  |  |  |  |
|  |  |  |  |  |  |  |  |  |  |  |  |  |
|  |  |  |  |  |  |  |  |  |  |  |  |  |
|  |  |  |  |  |  |  |  |  |  |  |  |  |
| Key: Y = Yes, N = No  Items: 1. Clearly defined question, 2. Consecutive or random sampling, 3. Avoided inappropriate exclusions, 4. Sample representative of intended target population, 5. Raters blinded to additional cues that were not part of test, 6. Assessor blinding during analyses, 7. Appropriate interval between successive tests, 8. All participants received all tests, 9. Successive tests performed under same conditions, 10. All participants included in analyses, 11. Appropriate statistical measures of agreement. | | | | | | | | | | | | |

**Additional file 3. Custom methodological quality checklist.**
