# Supplementary material for: Determining the number of stimuli required to reliably assess corticomotor excitability and primary motor cortical representations using transcranial magnetic stimulation (TMS): a protocol for a systematic review and meta-analysis
Source: Syst Rev. 2015 Aug 11;4:107. doi: 10.1186/s13643-015-0095-2 (PMC4531429; doi:10.1186/s13643-015-0095-2)
Supplement: Additional file 4: — TMS-specific methodology checklist. Checklist that will be utilised to assess the TMS-specific methodological and reporting quality of included studies. (DOCX 15 kb) [file 13643_2015_95_MOESM4_ESM.docx]

| **Controlled/Reported?** | Study | Study | Study | Study |
| --- | --- | --- | --- | --- |
| **Participant factors** |  |  |  |  |
| Age of subjects |  |  |  |  |
| Gender of subjects |  |  |  |  |
| Handedness of subjects |  |  |  |  |
| Subjects prescribed medication |  |  |  |  |
| Use of CNS active drugs (e.g. anti-convulsants) |  |  |  |  |
| Presence of neurological/psychiatric disorders |  |  |  |  |
| Any medical conditions |  |  |  |  |
| History of specific repetitive motor activity |  |  |  |  |
| **Methodological factors** |  |  |  |  |
| Position and contact of EMG electrodes |  |  |  |  |
| Amount of contraction of target muscles |  |  |  |  |
| Prior motor activity of the muscle to be tested |  |  |  |  |
| Relaxation of muscles other than those tested |  |  |  |  |
| Coil type (size and geometry) |  |  |  |  |
| Coil orientation |  |  |  |  |
| Direction of induced current in the brain |  |  |  |  |
| Coil location and stability |  |  |  |  |
| Type of stimulator used (e.g. brand) |  |  |  |  |
| Stimulation intensity |  |  |  |  |
| Pulse shape (monophasic or biphasic) |  |  |  |  |
| Determination of optimal hotspot |  |  |  |  |
| The time between MEP trials |  |  |  |  |
| Time between days of testing |  |  |  |  |
| Subject attention (level of arousal) during testing |  |  |  |  |
| Method for determining threshold (active/resting) |  |  |  |  |
| Number of MEP measures made |  |  |  |  |
| Method for determining MEP size during analysis |  |  |  |  |
| **Total Score /26** |  |  |  |  |

**Additional file 4. TMS-specific methodology checklist.**
